# Patterns of care and outcomes of patients with METAstatic soft tissue SARComa in a real-life setting: the METASARC observational study

**DOI:** 10.1186/s12916-017-0831-7

**Published:** 2017-04-10

**Authors:** Marion Savina, Axel Le Cesne, Jean-Yves Blay, Isabelle Ray-Coquard, Olivier Mir, Maud Toulmonde, Sophie Cousin, Philippe Terrier, Dominique Ranchere-Vince, Pierre Meeus, Eberhard Stoeckle, Charles Honoré, Paul Sargos, Marie-Pierre Sunyach, Cécile Le Péchoux, Antoine Giraud, Carine Bellera, François Le Loarer, Antoine Italiano

**Affiliations:** 1 Clinical and Epidemiological Research Unit, Institut Bergonié, Bordeaux, France; 2 ISPED, INSERM U1219 Bordeaux Population Health Center, Epicene Team, Bordeaux, France; 3 Department of Medicine, Institut Gustave Roussy, Villejui, France; 4 Department of Medicine, Centre Leon Berard, Lyon, France; 5 Department of Medicine, Institut Bergonié, Bordeaux, France; 6 Department of Pathology, Institut Gustave Roussy, Villejuif, France; 7 Department of Pathology, Centre Leon Berard, Lyon, France; 8 Department of Surgery, Centre Leon Berard, Lyon, France; 9 Department of Surgery, Institut Bergonié, Bordeaux, France; 10 Department of Surgery, Institut Gustave Roussy, Villejuif, France; 11 Department of Radiotherapy, Institut Bergonié, Bordeaux, France; 12 Department of Radiotherapy, Centre Leon Berard, Lyon, France; 13 Department of Radiotherapy, Institut Gustave Roussy, Villejuif, France; 14 Department of Pathology, Institut Bergonié, Bordeaux, France; 15 Early Phase Trials and Sarcoma Units, Institut Bergonié, 229 Cours de l’Argonne, Bordeaux, France

**Keywords:** Sarcoma, Metastases, Outcome, Patterns of care, Chemotherapy, Surgery

## Abstract

**Background:**

Well-designed observational studies of individuals with rare tumors are needed to improve patient care, clinical investigations, and the education of healthcare professionals.

**Methods:**

The patterns of care, outcomes, and prognostic factors of a cohort of 2225 patients with metastatic soft tissue sarcomas who were diagnosed between 1990 and 2013 and documented in the prospectively maintained database of the French Sarcoma Group were analyzed.

**Results:**

The median number of systemic treatments was 3 (range, 1–6); 27% of the patients did not receive any systemic treatment and 1054 (49%) patients underwent locoregional treatment of the metastasis. Half of the patients who underwent chemotherapy (n = 810) received an off-label drug. Leiomyosarcoma was associated with a significantly better outcome than the other histological subtypes. With the exception of leiomyosarcomas, the benefit of a greater than third-line regimen was very limited, with a median time to next treatment (TNT) and overall survival (OS) ranging between 2.3 and 3.7 months and 5.4 and 8.5 months, respectively. The TNT was highly correlated with OS. Female sex, leiomyosarcoma histology, locoregional treatment of metastases, inclusion in a clinical trial, and treatment with first-line polychemotherapy were significantly associated with improved OS in the multivariate analysis.

**Conclusions:**

The combination of doxorubicin with a second drug, such as ifosfamide, represents a valid option, particularly when tumor shrinkage is expected to provide clinical benefits. After failure of the second-line therapy, best supportive care should be considered, particularly in patients with non-leiomyosarcoma histology who are not eligible to participate in a clinical trial. Locoregional treatment of metastasis should always be included in the therapeutic strategy when feasible. TNT may represent a useful surrogate endpoint for OS in clinical studies.

**Electronic supplementary material:**

The online version of this article (doi:10.1186/s12916-017-0831-7) contains supplementary material, which is available to authorized users.

## Background

Soft-tissue sarcomas (STSs) represent a heterogeneous group of diseases that account for 1% of all malignancies in adults [1]. Despite adequate locoregional treatment, up to 40% of patients with STSs will develop metastatic disease [1, 2]. When metastases are detected, the standard of care is based on palliative chemotherapy. Due to their rarity, no specific data on the comprehensive management and outcomes of metastatic STS patients are available.

A national network of care coordinated by three national reference centres has been set up through the support of the French National Cancer Institute for the management of STS patients. All suspected or diagnosed STS cases are reviewed by an accredited pathologist who is an expert in the field, and the cases are included in a national database. The aim of this study was to use this unique set of data to assess the modalities of treatment of patients with metastatic STS in a real-life setting, to evaluate their impact on the outcome according to the histological subtype, and to identify prognostic factors.

## Methods

### Patients

From 1990 to 2013, patients ≥ 18 years old with a diagnosis of metastatic STS (excluding gastrointestinal stromal tumors, visceral sarcomas, and Ewing tumors) who were evaluated at one of the three national reference centres designated by the French National Cancer Institute for the management of STS (Centre Léon Bérard, Lyon; Institut Bergonié, Bordeaux; and Institut Gustave Roussy, Villejuif) were included in the prospectively maintained database of the French Sarcoma Group. A histological review of all patients was performed by the members of the pathological sub-committee of the French Sarcoma Group. The histological diagnosis and grading was established according to the World Health Organization Classification of Tumours and to the French grading system [2, 3].

### Outcomes

Time to next treatment (TNT) was defined as the time from the systemic treatment onset to the next treatment or death due to any cause, whichever came first. When neither death nor new systemic therapy was observed, TNT was censored at the date of last patient contact. Overall survival (OS) was defined as the interval between the diagnosis of metastatic disease or the first-line systemic therapy onset and the time of death. When death was not observed, OS was censored at the date of last patient contact.

### Statistical analysis

The statistical analysis of the baseline demographics and clinical outcomes was based on all data available up to the cut-off date of December 31, 2015. Descriptive statistics were used to show the distribution of variables in the population. Multivariate logistic regression models were used to identify biological and clinical factors associated with the type of treatment received and with the probability of survival 5 years after the diagnosis of metastases. Follow-up times were described as median values based on the inverse Kaplan–Meier estimator [4].

Prognostic factors of TNT and OS were identified using Cox proportional hazard models. The variables included in the univariate and multivariate analyses are detailed in Additional file 1.

The correlation between TNT and OS was evaluated at each of the four first-lines of metastatic chemotherapy by a Spearman rank correlation coefficient and was expressed as a value between 0 (no association) and 1 (perfect association). We used a reviewed copula-based approach that introduced an iterative multiple imputation method [5] for the estimation of the correlation coefficient. The data were analyzed using the SAS v9.3 and R v3.3 software packages.

## Results

### Patients

A total of 2165 patients were included in this study. Their characteristics are presented in Table 1. The median follow-up duration was 61 months (range, 1–300). The five most frequently detected histological subtypes were leiomyosarcoma (LMS), undifferentiated pleomorphic sarcoma (UPS), synovial sarcoma (SS), dedifferentiated liposarcoma (DLPS), and malignant peripheral nerve sheath tumors (MPNST).

**Table 1:** Patient characteristics according to the study population

	All patients		Patients alive at 5 years		Patients treated with metastatic chemotherapy	
	(n = 2165)		(n = 224)		(n = 1575)	
	n	%	n	%	n	%
Sex						
Male	1055	48.73	92	41.07	754	47.87
Female	1110	51.27	132	58.93	821	52.13
Age at first metastasis						
< 75 years old	1886	87.11	216	96.43	1429	90.73
≥ 75 years old	279	12.89	8	3.57	146	9.27
Histology						
Leiomyosarcoma	502	23.19	60	26.79	396	25.14
UPS	203	9.38	9	4.02	141	8.95
DLPS	172	7.94	12	5.36	112	7.11
Synovial sarcoma	188	8.68	16	7.14	150	9.52
MPNST	80	3.70	11	4.91	50	3.17
Other	1020	47.11	116	51.79	726	46.10
Grade						
1	138	6.37	48	21.43	94	5.97
2	590	27.25	74	33.04	440	27.94
3	1083	50.02	63	28.13	765	48.57
Not available	354	16.35	39	17.41	276	17.52
Number of metastatic sites						
1	1780	82.22	199	88.84	1248	79.24
> 1	385	17.78	25	11.16	327	20.76
Metastatic sites						
Lung	1399	64.62	149	66.52	1075	68.25
Liver	410	18.94	34	15.18	352	22.35
Peritoneum	396	18.29	60	26.79	319	20.25
Bone	370	17.09	29	12.95	305	19.37
Lymph node	304	14.04	35	15.63	236	14.98
Skin	172	7.94	25	11.16	136	8.63
Soft tissue	173	7.99	36	16.07	135	8.57
Pleura	163	7.53	11	4.91	140	8.89
Brain	113	5.22	5	2.23	89	5.65
Bone marrow	12	0.55	0	0.00	10	0.63
Other	228	10.53	32	14.29	166	10.54

*UPS* undifferentiated pleomorphic sarcoma, *DLPS* dedifferentiated liposarcoma, *MPNST* malignant peripheral nerve sheath tumors

### General treatment patterns

The general treatment patterns are described in Table 2. Patients over 75 years of age (*P* < 0.0001) and with MPNST (*P* = 0.0136) had a lower probability of receiving any systemic treatment, whereas presence of liver, lung, peritoneal, bone, pleural, skin, or lymphatic metastases was associated with a higher probability of receiving chemotherapy. Being over 75 years (*P* < 0.0001), DLPS (*P* = 0.0031), a grade 3 (*P* = 0.0188), and the presence of more than one metastatic site (*P* < 0.0001) were associated with a lower probability of receiving a locoregional treatment, whereas being a woman (*P* = 0.0012), SS (*P* = 0.0026), and the presence of lymphatic, brain, bone, skin, soft tissue, or peritoneal metastases were associated with an increased probability of locoregional treatment. Locoregional metastasis treatment was the sole treatment for 250 patients (11.55%). The metastasis localization was the only factor associated with the probability of receiving only locoregional treatment. Indeed, the presence of liver (*P* < 0.0001), lung (*P* < 0.0001), pleural (*P* = 0.0005), and peritoneal (*P* = 0.0087) metastases was associated with a lower probability of locoregional treatment alone, whereas patients with soft-tissue metastases (*P* = 0.0031) were more likely to receive only a locoregional treatment. Best supportive care alone was more likely to be proposed to patients over 75 years (*P* < 0.0001), with a grade 3 tumor (*P* = 0.0306), or with multiple metastatic sites (*P* = 0.0201).

**Table 2:** General patterns of treatment according to study population

	All patients		Patients alive at 5 years		Patients treated with chemotherapy	
	(n = 2165)		(n = 224)		(n = 1575)	
	n	%	n	%		
Metastatic treatment received						
Best supportive care only	340	15.70	13	5.80	0	0.00
Locoregional treatment	1054	48.68	187	83.48	804	51.05
Surgery	408	38.71	82	43.85	282	35.07
Radiotherapy	254	24.10	12	6.42	213	26.49
Radiofrequency	42	3.98	9	4.81	33	4.10
Other	30	2.85	3	1.60	19	2.36
Combination	320	30.36	81	43.32	257	31.97
None	1111	51.32	37	16.52	771	48.95
Chemotherapy	1575	72.75	156	69.64	1575	100
None	590	27.25	68	30.36	–	–
1 line	489	22.59	54	34.62	489	31.05
2 lines	293	13.53	24	15.38	293	18.60
3 lines	240	11.09	21	13.46	240	15.24
4 lines	157	7.25	11	7.05	157	9.97
> 4 lines	396	17.27	46	29.49	396	25.15
Anthracycline received						
Yes	–	–	109	69.87	951	60.38
No	–	–	47	30.13	624	39.62
Anthracycline received as first line						
Yes	–	–	98	62.82	852	54.10
No	–	–	58	37.18	723	45.90
Polychemotherapy received as first line						
Yes	–	–	95	60.90	716	45.46
No	–	–	61	39.10	859	54.54
Inclusion in a clinical trial						
Yes:	–	–	55	35.26	332	21.08
Line 1	–	–	10	6.41	122	7.75
Line 2	–	–	17	16.67	107	9.85
Line 3	–	–	10	12.82	56	7.06
Line 4	–	–	7	12.28	30	5.42
Other lines	–	–	11	23.91	17	4.29
No	–	–	101	64.74	1243	78.92
Off-label drugs						
Yes:	–	–	99	63.46	810	51.43
Line 1	–	–	21	13.46	194	12.32
Line 2	–	–	22	21.57	203	18.69
Line 3	–	–	14	17.95	169	21.31
Line 4	–	–	21	36.84	142	25.68
Other lines	–	–	21	45.65	102	25.76
No	–	–	57	36.54	765	48.57

### Systemic treatment patterns (Table 2)

The median number of systemic treatments received by the patients was 3 (min = 1 and max = 6) and did not significantly differ across the histological subtypes. Patients < 75 years old (*P* < 0.0001) and those with lymph node involvement (*P* = 0.0001) were more likely to receive polychemotherapy in the first-line setting. The most frequently prescribed off-label drug was gemcitabine. Female sex (*P* = 0.0313) and age ≥ 75 years (*P* = 0.0003) were factors associated with a lower probability of being part of a clinical trial. On the contrary, patients with LMS or SS (*P* = 0.0217) and patients with liver (*P* = 0.0072), skin (*P* = 0.0013) or peritoneal (*P* = 0.0036) metastases were more likely to be included in a clinical trial during the course of their treatment.

### Time to next treatment and overall survival

The median TNT and OS according to the treatment line setting for the five most frequent histological subtypes are described in Table 3. Patients with metastatic LMS had the longest median survival, whereas patients with UPS had the shortest. The benefit of systemic therapy beyond the second line setting was limited, with a median TNT ranging between 2.3 and 3.5 months except for LMS (>4 months). The correlation estimated between TNT and OS was similar and high regardless of the considered chemotherapy line (rho > 0.65); the highest value was observed in the first line setting (rho = 0.76; 95% CI, 0.73–0.78) (Table 4).

**Table 3:** Median time to next treatment (TNT) and overall survival (OS) according to the histological subtype and treatment setting

	Median TNT/OS (months)			
	TNT1/OS1^a^	TNT2/OS2^b^	TNT3/OS3^c^	TNT4/OS4^d^
LMS	8.0/24.9	5.6/17.3	4.6/12.3	4.4/9.2
UPS	4.8/11.0	3.5/7.9	2.3/3.7	3.5/6.2
DLPS	4.4/11.8	5.1/8.8	2.4/6.0	3.2/8.5
SS	8.7/19.7	5.7/11.7	3.4/7.8	2.3/6.0
MPNST	4.1/12.5	2.8/7.0	3.6/8.0	3.7/5.4

^a^Calculated from the date of first-line treatment onset

^b^Calculated from the date of second-line treatment onset

^c^Calculated from the date of third-line treatment onset

^d^Calculated from the date of fourth-line treatment onset

*DLPS* dedifferentiated liposarcomas, *LMS* leiomyosarcomas, *MPNST* malignant peripheral nerve sheath sarcomas, *SS* synovial sarcomas, *UPS* undifferentiated pleomorphic sarcomas

**Table 4:** Correlation between time to next treatment (TNT) and overall survival (OS)

	Spearman’s rho	95% CI
TNT1/OS1^a^	0.76	0.73–0.78
TNT2/OS2^b^	0.70	0.67–0.73
TNT3/OS3^c^	0.68	0.65–0.72
TNT4/OS4^d^	0.73	0.70–0.76

^a^Calculated from the date of first-line treatment onset

^b^Calculated from the date of second-line treatment onset

^c^Calculated from the date of third-line treatment onset

^d^Calculated from the date of fourth-line treatment onset

### Prognostic factors for time to next treatment

We evaluated the prognostic TNT value calculated from the first line systemic therapy of the main biological, histological, and clinical factors for the 1575 patients who received at least one systemic treatment (Table 5).

**Table 5:** Prognostic factors for time to next treatment

	Univariate analysis		Multivariate analysis	
Covariate	*P*	HR (95% CI)	*P*	HR (95% CI)
Sex (ref: Male)	0.0014	0.835 (0.747–0.933)	0.0013	0.825 (0.733–0.928)
Age (ref: < 75 years old)	0.0023	1.374 (1.120–1.686)	–	–
Histotype (ref: Other)				
LMS	0.5114	0.955 (0.831–1.097)	–	–
DLPS	0.0068	1.357 (1.088–1.692)	–	–
MPNST	0.3703	1.154 (0.843–1.580)	–	–
SS	0.8580	0.983 (0.811–1.191)	–	–
UPS	0.0375	1.243 (1.013–1.525)	–	–
Grade (ref: < 3)	< 0.0001	1.417 (1.258–1.596)	< 0.0001	1.372 (1.218–1.546)
Number of metastatic sites (ref: 1)	0.1175	1.118 (0.972–1.285)	–	–
Liver metastasis (ref: no)	0.1436	1.103 (0.967–1.259)	–	–
Locoregional treatment (ref: no)	< 0.0001	0.496 (0.442–0.556)	< 0.0001	0.487 (0.432–0.550)
Clinical trial in first line (ref: no)	0.6453	1.048 (0.859–1.277)	–	–
Anthracycline in first line (ref: no)	< 0.0001	0.756 (0.674–0.847)	–	–
Polychemotherapy in first line (ref: no)	< 0.0001	0.729 (0.651–0.815)	< 0.0001	0.743 (0.660–0.836)

*DLPS* dedifferentiated liposarcomas, *LMS* leiomyosarcomas, *MPNST* malignant peripheral nerve sheath sarcomas, *SS* synovial sarcomas, *UPS* undifferentiated pleomorphic sarcomas

**Fig. 1:**
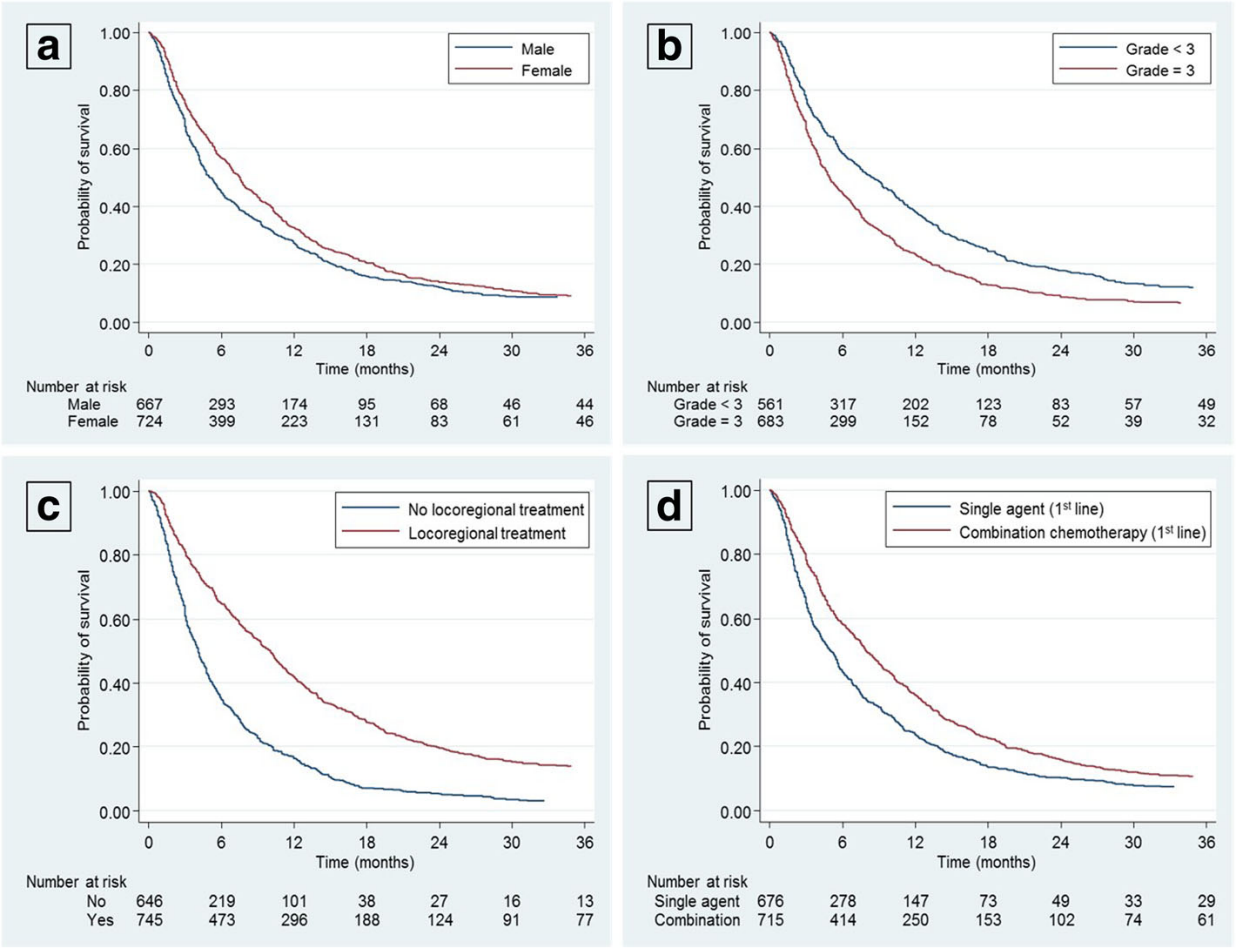
Prognostic factors of time to next treatment – Kaplan–Meier curves. Kaplan-Meier Curves of time to next treatment according to (**a**) gender, (**b**) grade, (**c**) locoregional treatment of metastases, and (**d**) type of systemic treatment

Regarding the multivariate analysis, the following factors remained associated with an increased TNT: female sex, locoregional treatment of metastases, and administration of polychemotherapy in the first line of metastatic treatment (Table 5, Fig. 1). Only a grade 3 tumor at diagnosis remained associated with a decreased TNT (Table 5, Fig. 1).

### Prognostic factors for OS

We evaluated the prognostic OS values of the main biological, histological, and clinical factors for the 1575 patients who received at least one systemic treatment (Table 6).

**Table 6:** Prognostic factors for overall survival

	Univariate analysis		Multivariate analysis	
Covariate	*P*	HR (95% CI)	*P*	HR (95% CI)
Sex (ref: Male)	0.0002	0.801 (0.713–0.899)	0.0003	0.792 (0.698–0.900)
Age (ref: < 75 years old)	0.0024	1.389 (1.123–1.717)	–	–
Histotype (ref: Other)				
LMS	0.0004	0.765 (0.659–0.888)	0.0010	0.765 (0.652–0.897)
DLPS	0.0269	1.291 (1.030–1.619)	0.2034	1.171 (0.918–1.492)
MPNST	0.1368	1.273 (0.926–1.751)	0.2183	1.234 (0.883–1.726)
SS	0.4738	1.074 (0.883–1.307)	0.0764	1.206 (0.980–1.485)
UPS	0.0061	1.347 (1.089–1.668)	0.1839	1.168 (0.929–1.469)
Grade (ref: < 3)	< 0.0001	1.692 (1.491–1.920)	< 0.0001	1.687 (1.483–1.919)
Number of metastatic sites (ref: 1)	0.0136	1.200 (1.038–1.387)	0.0009	1.305 (1.115–1.528)
Liver metastasis (ref: no)	0.1056	0.891 (0.774–1.025)	–	–
Locoregional treatment (ref: no)	< 0.0001	0.412 (0.365–0.465)	< 0.0001	0.400 (0.351–0.455)
Clinical trial (ref: no)	< 0.0001	0.750 (0.653–0.862)	0.0002	0.755 (0.651–0.877)
Off-label drugs (ref: no)	< 0.0001	0.791 (0.703–0.890)	–	–
Anthracycline (ref: no)	0.0046	0.838 (0.741–0.947)	–	–
Anthracycline in first line (ref: no)	0.0127	0.861 (0.765–0.968)	–	–
Polychemotherapy in first line (ref: no)	0.0003	0.804 (0.715–0.902)	0.0023	0.822 (0.724–0.932)

*DLPS* dedifferentiated liposarcomas, *LMS* leiomyosarcomas, *MPNST* malignant peripheral nerve sheath sarcomas, *SS* synovial sarcomas, *UPS* undifferentiated pleomorphic sarcomas

**Fig. 2:**
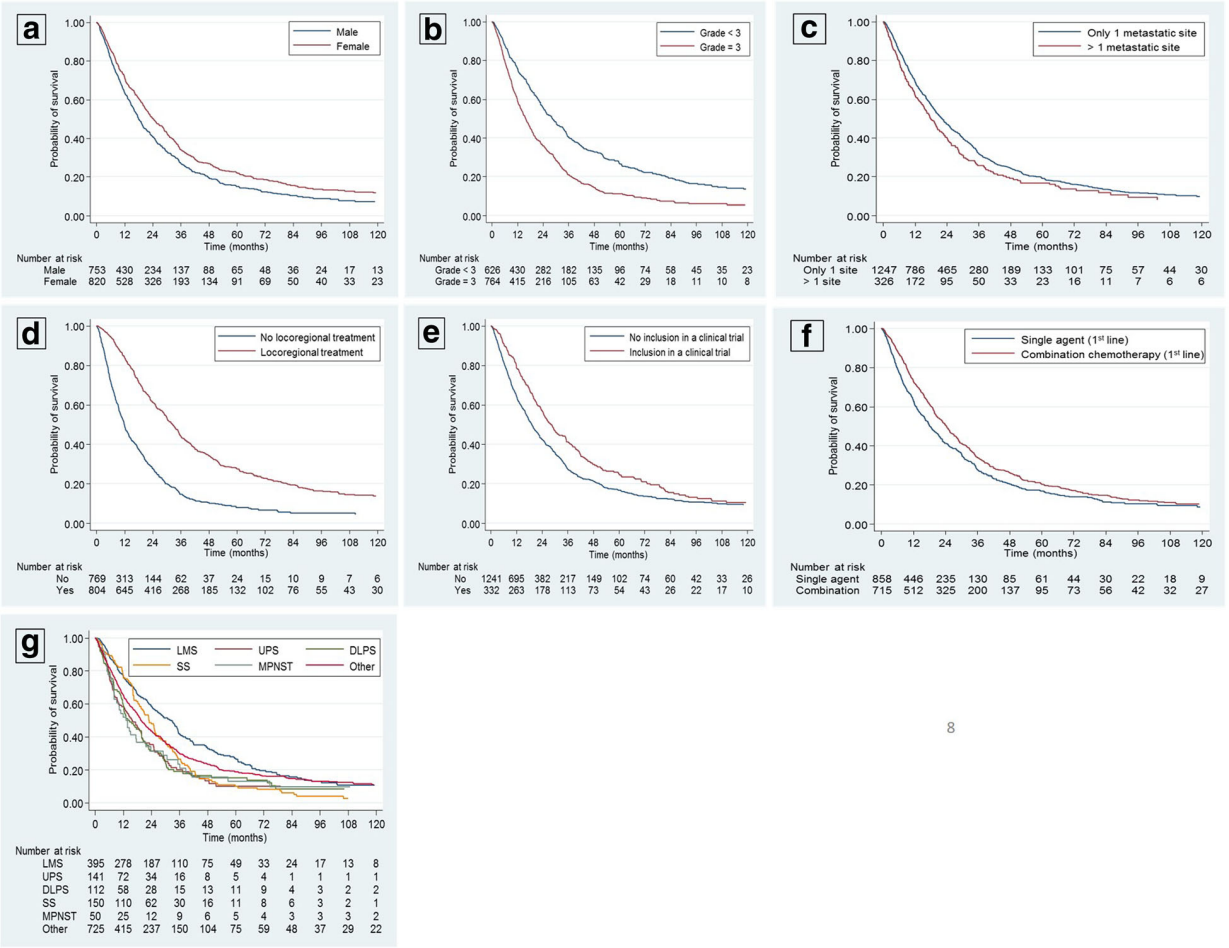
Prognostic factors of overall survival – Kaplan–Meier curves. Kaplan-Meier curves of Overall survival according to (**a**) gender, (**b**) grade, (**c**) number of metastatic sites, (**d**) locoregional treatment of metastases, (**e**) inclusion in a clinical trial, (**f**) type of systemic treatment, (**g**) histological subtype

The following factors remained associated with an increased OS in the multivariate analysis: female sex, LMS, locoregional treatment of metastases, inclusion in a clinical trial, and administration of polychemotherapy in the first line of metastatic treatment (Table 6, Fig. 2). A grade 3 tumor at diagnosis remained associated with a decreased OS (Table 6, Fig. 2).

### Parameters correlated with 5-year survival

To evaluate the parameters associated with a long survival, we excluded patients alive and with a follow-up inferior to 5 years, leading to the inclusion of 1619 patients in this analysis. A total of 224 patients were alive 5 years after the diagnosis of metastasis. The characteristics and patterns of this population are described in Tables 1 and 2, respectively.

**Fig. 3:**
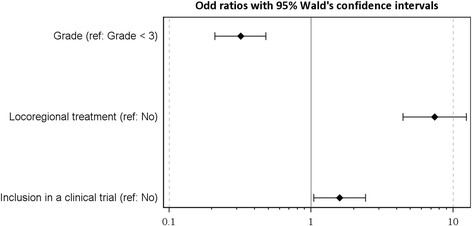
Prognostic factors for 5-year survival – Odd ratios with 95% Wald’s confidence intervals

The odds ratios and confidence intervals estimated by the logistic regression model for the factors significantly associated with the probability of 5-year survival are presented in Fig. 3. The factors associated with a higher probability of 5-year survival were locoregional treatment of metastases (OR = 7.41; 95% CI, 4.42–12.41) and inclusion in a clinical trial (OR = 1.59; 95% CI, 1.04–2.42). A grade 3 tumor at the time of diagnosis of metastasis was associated with a lower probability of 5-year survival (OR = 0.32; 95% CI, 0.21–0.48).

To observe the impact of the locoregional treatment modality on the probability of 5-year survival, we replaced the binary variable “locoregional treatment: yes/no” by a categorical variable detailing the type of locoregional treatment received (surgery, radiotherapy, radiofrequency, other, combination, or none). The following locoregional treatment modalities were particularly and significantly associated with a higher probability of 5-year survival: surgery (OR = 11.20; 95% CI, 6.19–20.26), radiofrequency (OR = 15.62; 95% CI, 5.04–48.41), and combination of modalities (OR = 9.60; 95% CI, 5.38–17.14). Other types of treatment, such as radiotherapy, were also correlated with a better probability of long survival; however, the effect was not significant.

## Discussion

The heterogeneity of STS has rarely been taken into account in the design of clinical trials to investigate systemic therapies in STS patients. Our results indicated that LMS clearly represented a distinct STS subgroup with a significantly better outcome in the advanced setting. Previous studies have shown worse outcomes for LMS than the results obtained in our current analysis. The largest study published to date was a retrospective analysis of 2185 patients with advanced STS treated in the first-line studies of EORTC-STBSG; these patients showed no significant differences in terms of OS between LMS (492 cases) and the other histological subtypes, with a median OS of approximately 12 months [6]. However, this study, which focused only on first-line treatment, included patients diagnosed before the identification of the KIT mutation in gastrointestinal stromal tumors [4]. Therefore, a significant proportion of gastrointestinal stromal tumors, which are chemorefractory, were likely included in the LMS group. The better outcome of LMS may be explained by a specific biology but also by the potentially higher sensitivity to some anti-cancer agents such as gemcitabine, dacarbazine, or trabectedin. For instance, in a recent phase II randomized trial, patients with leiomyosarcomas of any origin benefited significantly from the combination of gemcitabine with dacarbazine, achieving a median progression-free survival (PFS) and OS of 4.9 and 13.8 months, respectively, versus 2.1 and 7.8 months, respectively, for the non-leiomyosarcoma subtypes [7]. Moreover, a large worldwide expanded access program for trabectedin showed a median OS of 16.2 months in 321 heavily pre-treated leiomyosarcoma patients versus a median survival time of 11.9 months for the whole cohort of 903 patients [8].

We report here the first study assessing the outcomes of patients with advanced UPS. Some past reports included patients with malignant fibrous histiocytomas (MFHs). However, a significant subset of tumors initially diagnosed as MFH showed a specific line of differentiation (lipogenic, neurogenic, myogenic, or non-sarcomatous) [9–12]. “MFH” is now considered an obsolete terminology and has been replaced by the term UPS, which is a diagnosis of exclusion. We found that patients with advanced UPS had the worst outcome with the shortest TNT and a median OS of only 11 months. These results illustrate the particular resistance to chemotherapy of this histological subset and an intrinsically more aggressive biology. Further investigations are needed to better understand the mechanisms of their tumorigenesis and to define more appropriate therapeutic strategies.

Approximately 45% of the 1575 patients who underwent systemic therapy received a combination chemotherapy regimen in the first-line setting. The first-line chemotherapy for advanced, metastatic, or non-resectable STS is typically based on single-agent doxorubicin [13]. Indeed, the majority of clinical studies comparing single agents with combinations failed to show an OS advantage but consistently showed improvement in the response rates and PFS [14, 15]. Interestingly, our analysis showed a significant impact of the use of combination chemotherapy on OS, with a hazard ratio of 0.822 (0.724–0.932) and *P* = 0.0003. Judson et al. [14] recently published the results of a randomized clinical trial evaluating doxorubicin as a single agent in the control arm versus doxorubicin-ifosfamide in the experimental arm as a first-line treatment for advanced or metastatic STS. Although the Kaplan–Meier curves presented in the publication highlighted a difference between the two treatment arms in favor of polychemotherapy, the trial failed to detect a significant effect of polychemotherapy on OS, which was in contrast to our results. Our results suggest that the negative outcome of this study may simply be due to a lack of power as already suggested by Benjamin and Lee [16]. Indeed, by including 450 patients and observing at least 366 events, the trial was designed to detect a maximum HR of 0.737. Due to the large size of our dataset, we were able to observe an HR of 0.822. Based on their hypotheses, a total of 827 events would be required to detect a similar treatment effect in a randomized clinical trial. Although our study suggests a benefit in terms of OS, clinicians should also be aware that randomized trials have clearly demonstrated that combination chemotherapy is more toxic than single-agent doxorubicin with a potential significant impact on the quality of life [14, 15]. Therefore, a combination of doxorubicin with a second drug such as ifosfamide should be used only after a careful discussion with the patient on the benefit/risk ratio of this approach, particularly when tumor shrinkage is expected to improve the symptoms or clinical benefits.

A high proportion of patients received more than two lines of systemic treatment. With the exception of leiomyosarcomas, our results indicate that the benefit of a greater than third-line regimen is very limited, with the median TNT and OS ranging between 2.3 and 3.7 months and 5.4 and 8.5 months, respectively. This result is consistent with the data from the PALETTE study, which led to the approval of pazopanib in advanced STS [17]. In that study, the number of previous lines of chemotherapy was a significant prognostic factor in the multivariate analysis for PFS with a significantly worse outcome in patients receiving pazopanib in the third- or fourth-line settings versus the first- or second-line settings. Given the potential toxicity and the moderate benefit of systemic therapy after failure of the second-line treatment, best supportive care should be considered as a reasonable option, particularly in patients with non-leiomyosarcoma histology and a poor performance status or patients who were not eligible to participate in a clinical trial. Notably, 50% of patients received an off-label drug during their treatment disease course. This result reflects the increasing evidence for the use of other drugs besides doxorubicin and ifosfamide in the sarcoma field. The most frequently prescribed off-label drug in this study was gemcitabine. Indeed, gemcitabine with or without docetaxel is commonly used in some specific sarcoma subsets, particularly in leiomyosarcomas and angiosarcomas [18–21], although neither of these drugs is approved for this indication. Another not yet approved drug that is frequently used in the sarcoma field is paclitaxel, which shows activity particularly in angiosarcomas [22, 23].

A significant proportion of patients with metastatic STS (27%) did not receive any systemic therapy. An age > 75 years was significantly associated with a lower probability of receiving any systemic treatment. Aging is associated with progressive functional declines, an increased prevalence of comorbidities, and a higher risk of cardiac and hematological toxicities related to anthracyclines [24–26]. These data may explain the reluctance of oncologists to use chemotherapy in elderly patients with STS and raises the question of the development of adapted chemotherapy regimens for elderly patients with advanced STS, such as low-dose cyclophosphamide [27] or liposomal doxorubicin [28].

A total of 49% of the patients received a loco-regional treatment of the metastasis, the most frequent of which were surgery followed by radiotherapy and radiofrequency ablation. The majority of these patients (71%) had lung metastases. The published evidence on the role of locoregional treatments, such as pulmonary metastasectomy, is derived from a small number of studies with limited sample sizes [29]. Primary bone sarcomas, which may represent a distinct disease, are often included in these analyses. Our present study differed from previous publications because we used a larger database cohort, which increased the power of the multivariate analysis; additionally, we focused on STS exclusively to enhance the homogeneity of the study population. As suggested by previous studies, patients who underwent a locoregional metastasis treatment had improved survival in the multivariate analysis. Arguments have suggested that an observational study may not provide evidence that a difference in survival is attributable to the locoregional treatment and that only a randomized trial can answer the question. However, we observed that more than 80% of metastatic patients alive 5 years after the diagnosis of metastasis had received a locoregional treatment, versus 50% in the general population, and this parameter was most significantly associated with the probability of being alive at 5 years in the logistic regression model. Precisely, the descriptive analyses of the patients alive after 5 years suggest that surgery, radiofrequency, and a combination of different modalities are particularly beneficial in terms of survival. This hypothesis was confirmed by our sensibility analysis, since we found that the positive effect on the probability of 5-year survival was significant for these three treatment modalities only.

No data are available from randomized clinical trials to define how best to integrate the locoregional treatment of metastases in the management of patients with advanced disease. The most recent attempts were made by the European Organisation for Research and Treatment of Cancer (EORTC-Protocol 62933) with a randomized multicenter trial to assess metastasectomy alone versus induction chemotherapy followed by metastasectomy in a targeted sample size of 340 patients. Started in 1996, this trial was closed due to poor accrual in November of 2000. Notably, we report here the first large series of patients who received non-surgical locoregional treatment of metastases, including 254 patients treated with radiotherapy, 42 with radiofrequency ablation, and 320 with a combination of surgery plus radiotherapy or surgery plus radiofrequency ablation of metastases.

The gold standard endpoint in randomized clinical trials in oncology is OS. However, the use of a surrogate endpoint at an earlier stage in clinical trials would speed up the assessment of treatments and might reduce the cost of drug development. Studies that assess the use of alternative outcome measures, such as the response rate or PFS, as surrogate endpoints for OS in sarcoma patients showed only a modest if any correlation with PFS and OS [30, 31]. This issue was recently illustrated with the pivotal trial that led to eribulin approval in patients with liposarcomas that showed a benefit in OS but not in PFS [32]. TNT is an established endpoint that is mostly applied in hematological malignancies and has recently been used in breast, colon, and prostate cancer [33–35]. The use of this parameter is predicated on the concept that a change in treatment usually occurs in response to a real change in the patient status by integrating the efficacy and toxicity components. In our study, we found a strong correlation between TNT and OS. The prospective validation of this endpoint as a surrogate for OS should be done in future studies.

## Conclusions

This study reports the most comprehensive information related to the patterns of care and outcome of STS with advanced disease managed in the real-life setting. Limitations include its observational nature, which provides a lower level of evidence than a conventional clinical trial, the lack of data related to visceral sarcomas and GIST, and to the safety of therapeutic interventions. However, there are several lines of evidence indicating that observational studies usually do provide valid information and could be used to exploit well-designed databases [36].

## Additional file

Additional file 1: Supplementary Methods. (DOCX 11 kb)

## Abbreviations

DLPS: Dedifferentiated liposarcoma
LMS: Leiomyosarcoma
MFHs: Malignant fibrous histiocytomas
MPNST: Malignant peripheral nerve sheath tumors
OS: Overall survival
PFS: Progression-free survival
SS: Synovial sarcoma
STSs: Soft-tissue sarcomas
TNT: Time to next treatment
UPS: Undifferentiated pleomorphic sarcoma
