# Supplementary material for: Patterns of care and outcomes of patients with METAstatic soft tissue SARComa in a real-life setting: the METASARC observational study
Source: BMC Med. 2017 Apr 10;15:78. doi: 10.1186/s12916-017-0831-7 (PMC5385590; doi:10.1186/s12916-017-0831-7)
Supplement: Additional file 1: — Supplementary Methods. (DOCX 11 kb) [file 12916_2017_831_MOESM1_ESM.docx]

**Additional file 1: Methods**

Univariate and multivariate analyse for TNT and OS included the following biological and histological variables (Cox proportional hazard models): gender (female/male), age at diagnosis (under 75 years old/over 75 years old), histological subtype (leiomyosarcoma/undifferentiated pleomorphic sarcoma/dedifferentiated liposarcoma/synovial sarcoma/malignant peripheral nerve sheath tumour/other), grade (Grade 3/Grade <3), number of metastatic sites (>1 metastatic site/1 metastatic site) and the presence of liver metastases (yes/no). The following parameters related to the treatment received were also included in the models: locoregional treatment of metastases (yes/no), anthracycline administered (yes/no), anthracycline administered in the 1st line setting (yes/no), polychemotherapy administered in the 1st line setting (yes/no), off-label drugs administered (yes/no) and inclusion in a clinical trial (yes/no). Locoregional treatment of metastasis was defined as any procedure of local therapy for metastatic disease (i.e. surgery, radiotherapy, cryosurgery laser and microwave ablation) used alone or in combination. Off-label was defined as the use of anti-cancer drugs despite the absence of approval by a regulatory authority for the management of advanced sarcoma patients. .Factors associated with the outcome with a p-value <0.20 according to the univariate analysis (type one error of 20%) were included in the multivariate model. Then, the factors were selected using an ascending–descending stepwise model with a type one error of 10% for inclusion in the model and 5% for remaining in the model. The factors associated with the outcome with a p-value <0.10 (type-one error of 10%) in the multivariate model were individually included. At each step of the model, all included variables were tested and removed if they were no longer associated with the outcome considering a 5% type one error (p-value ≥0.05). All statistical tests were two-sided.
